# A Scoping Review on the Prevalence and Determinants of Post-Traumatic Stress Disorder among Military Personnel and Firefighters: Implications for Public Policy and Practice

**DOI:** 10.3390/ijerph19031565

**Published:** 2022-01-29

**Authors:** Gloria Obuobi-Donkor, Folajinmi Oluwasina, Nnamdi Nkire, Vincent I.O. Agyapong

**Affiliations:** 1 Department of Psychiatry, University of Alberta, Edmonton, AB T6G 2B7, Canada; folajinm@ualberta.ca (F.O.); Nnamdi.Nkire@albertahealthservices.ca (N.N.); agyapong@ualberta.ca (V.I.A.); 2 Department of Psychiatry, Dalhousie University, Halifax, NS B3H 2E2, Canada

**Keywords:** military personnel, firefighters, PTSD, prevalence, determinants

## Abstract

Introduction: Firefighters and military personnel are public safety personnel who protect the safety of individuals and their properties. They are usually exposed to traumatic events leaving them at risk of developing mental health conditions such as post-traumatic stress disorder (PTSD). Increasing concern is being raised regarding the mental health impacts, specifically PTSD, among military personnel and firefighters. Objective: There is an increased exposure of firefighters and military personnel to traumatic events and the attendant risk of developing post-traumatic stress disorder. It is crucial to ascertain the level of PTSD amongst this cohort and determinants to formulate policies and practices that mitigate the risk and protect public safety personnel. This scoping review sought to determine the prevalence of PTSD among this cohort globally and to explore determinants of this mental health condition. Methods: A literature search in databases including MEDLINE, CINAHL, PubMed, PsycINFO, and EMBASE was conducted electronically from May 2021 to 31 July 2021. Two reviewers independently assessed full-text articles according to the predefined inclusion criteria and screening process undertaken to identify studies for the review. Articles were screened with a third reviewer, resolving conflicts where necessary and further assessing them for eligibility. During article selection, the PRISMA checklist was adopted, and with the Covidence software, a total of 32 articles were selected for the final examination. For the eligible studies, data extraction was conducted, information was collated and summarized, and the findings were reported. Original qualitative and quantitative data on the prevalence and predictors of PTSD among veterans, military, and firefighters were reported. Results: The prevalence of PTSD was 57% for firefighters and 37.8% for military personnel. Demographic factors, job factors, social support, injuries, physical and psychological factors, and individual traits were the main predictors of PTSD in this cohort. Conclusion: This information is vital for developing and implementing prevention and intervention strategies for PTSD in military personnel and firefighters. Recognizing and addressing factors that predict PTSD will help to improve mental wellbeing and increase productivity. More peer-reviewed studies are needed on the prevalence of PTSD amongst these cohorts.

## 1. Introduction

Post-traumatic stress disorder (PTSD) is a mental disorder commonly reported among military personnel and firefighters [1]. This condition is usually chronic and may affect all aspects of life. Firefighters and military personnel fall into the category of public safety personnel (PSP) [2] due to their job description. They face many traumatic situations due to the nature of their work, and in the process of saving lives and property, they may sustain injuries and endanger their own lives [3]. This is amplified for military personnel deployed to war zones, who are exposed to a range of traumatic events, such as exposure to explosives [4]. Firefighters are usually the first point of call in domestic emergencies, such as fires, and are known as first responders. They save people’s lives, confront injuries and death associated with natural and artificial disasters, at times under the threat of personal injury, and prevent death or attempt to limit the damage. These potentially traumatic events are essential and set firefighters apart from other first responders, and they pose a significant psychological burden in this field of work [5,6,7].

PTSD can coexist with other conditions. In addition to PTSD, firefighters and military personnel are significantly at risk of experiencing other mental health conditions such as depression and anxiety due to the nature of their job [4,8,9]. The Diagnostic and Statistical Manual of Mental Disorders, 5th Edition (DSM-5), defined a traumatic event as experiencing, witnessing, or being confronted with at least one occasion of severe injury, threatened death, or sexual violence. The prevalence of PTSD may be influenced by occupation and the nature and severity of continuous exposure to traumatic events [10]. PTSD rates are very high among PSPs, including firefighters and military personnel [2]. This ranges from one-third to more than one-half of those exposed to potentially traumatic events [9]. The National Fallen Firefighters Foundation (NFFF) of the US shows that due to the diversity of the job description of other first responders, traumatic events differ, as does exposure to PTSD [11]. The prevalence of PTSD in combat veterans is estimated to be between 10% and 15%, with lifetime prevalence estimates ranging from 12% to 30% [12]. A critical review by Richardson et al. (2010) in the US and UK revealed that the point prevalence rate of PTSD for military personnel ranges from 2% to 17% [13]; the US military personnel returning from Afghanistan or Iraq recorded a higher prevalence of PTSD as compared to the UK military personnel returning from Afghanistan or Iraq [14].

Globally it is estimated that 10% to 35% of first responders, including firefighters, experience mental disorders [10,15]. A meta-analysis examining mental disorders among ambulance personnel found estimated prevalence rates of 11% for PTSD, 15% for depression, 15% for anxiety, and 27% for general psychological distress [16]. The study showed that PTSD is common among PSPs [16]. A study by Durham, McCammon, and Allison (1985) revealed 80% of rescue, firefighter, medical, and police personnel who treated victims of an apartment building explosion reported at least one symptom of PTSD [17,18]. The prevalence figures of PTSD in these groups were found to vary widely from 0% [19] to 46% [20]. Prevalence rates of PTSD symptoms of firefighters have ranged from 6.5% to 37% [21]. The significant variance in the prevalence rates of PTSD symptoms may be explained in the research population that is examined. When examining firefighters, military personnel, police, and paramedics specifically; PSPs in general; or a mixture of the individual professions, the sample sizes and selection of PTSD measures influence the results.

Researchers exploring the prevalence of PTSD in military personnel and firefighters have examined specific predictors and risk factors to better understand the values; amongst the commonly studied variables are gender, previous psychiatric history, and age [21,22]. The National Vietnam veterans Readjustment Study (NVVRS) selected 3016 American veterans as representative of personnel who serviced in the armed forces during the Vietnam period. The estimated lifetime prevalence rates of PTSD among these veterans were 30.9% and 26.9% for men and women, respectively [23].

The World Health Organization (WHO) completed an epidemiological study among 200,000 respondents in 27 countries [10]. The first 17 countries completed the World Mental Health Surveys. The results estimated lifetime PTSD prevalence ranges, from a low of 0.3% in China to 6.1% in New Zealand [23]. The PTSD rates found in the rescue teams ranged from 5% to 32% [24], with firefighters having a 21% rate [24]. However, the prevalence rates of PTSD for firefighters in the USA ranged from 8% to 22% [21,25]. The Canadian Community Health Survey of 2012 suggested a prevalence rate of PTSD of 1.7% among the Canadian population and 17% among firefighters [2], while male firefighters recorded a 20% rate of PTSD [26,27]. A study conducted among 677 individuals experiencing different types of traumas in Los Angeles revealed that 31% met the criteria for PTSD [28]. Notwithstanding, a meta-analysis reported a 7% prevalence of PTSD among firefighters [5]. A study on the mental health of firefighters in 16 provinces in China found 11% of firefighters’ mental well-being was poorly catered for, with 5% not cared for at all. Firefighters and military personnel experiencing PTSD may experience decreased productivity, increased risk of suicide, and poor social interactions [29]. Canada lost about 1.3% of its Gross Domestic Product (GDP) due to reduced labor output among workers with PTSD [2].

PTSD may co-occur with other mental illnesses or manifest symptoms similar to other mental illnesses. This may cause it to be misdiagnosed, resulting in the risk of appropriate and adequate treatment not being administered [29]. Determining the prevalence and determinants of PTSD among military personnel and firefighters will support the health and improve the quality of life in this cohort. To the best of our knowledge, this is the first scoping review article to examine the prevalence and determinants of PTSD in military personnel and firefighters. Epidemiological studies, whilst useful in this context, do not specifically explore all of the different determinants of PTSD, nor the prevalence of PTSD in this population. Thus, this scoping review was conducted to synthesize the data on PTSD regarding its relation to military personnel and firefighters. Specifically, we aimed to explore the literature related to the prevalence and determinants of PTSD among these PSPs.

## 2. Methods

This review was designed and conducted in adherence with the Preferred Reporting Items for Systematic Reviews and Meta-Analyses Extension for Scoping Reviews (PRISMA-ScR) statement [30]. This scoping review followed Arksey and O’Malley’s five-stage approach to scoping reviews [31].

A literature search was conducted in five databases, including PubMed, MEDLINE, PsycINFO, CINAHL, and EMBASE. A comprehensive review was completed, including articles from January 1985 to August 2021. Relevant and current articles were extracted, reviewed, and analyzed. Articles were screened with an overall goal of finding a group of articles that focused explicitly on PTSD among firefighters and military personnel. Qualitative and quantitative studies were included in this review. Furthermore, editorial, opinion, and theoretical articles were excluded from this review. All included papers were published in peer-reviewed journals.

## 3. Inclusion and Exclusion Criteria

Studies were considered eligible when they identified the prevalence of PTSD among firefighters, veterans, or military personnel from relevant journals. All articles were published in the English language.

We excluded articles if they did not focus on PTSD among firefighters or military personnel or did not identify the predictors of PTSD and its prevalence. Grey literature was not scoped, as some authors indicate that searching grey literature requires large investments of time, yields very little relevant material, and is not often considered appropriate by researchers [32]. In addition, only full texts in English were reviewed.

**Figure 1:**
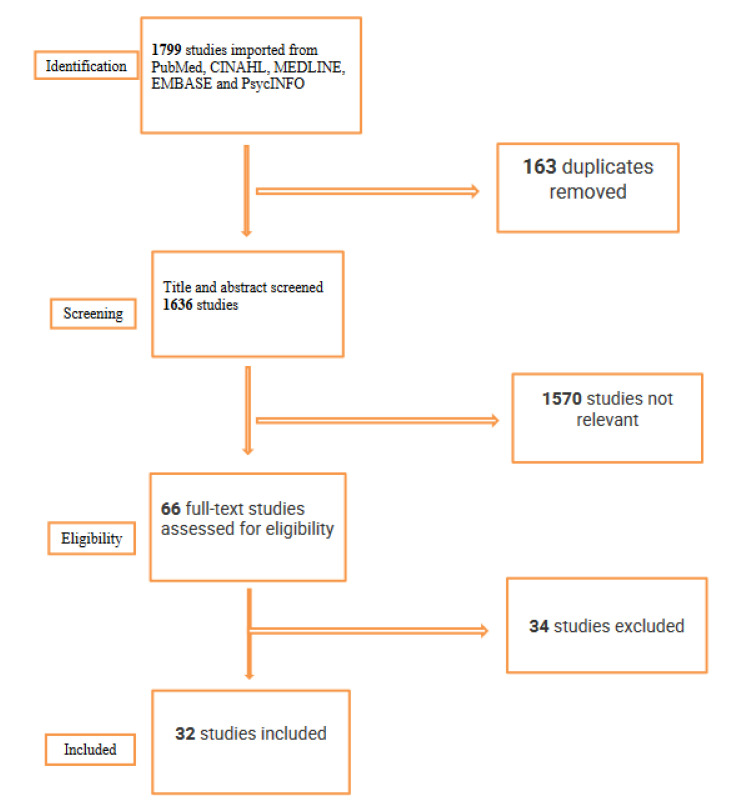
PRISMA flow chart.

The search strategy embraced a combination of MeSH terms, keywords, and descriptors including (PTSD OR post-traumatic stress disorder OR traumatic stress disorder OR stress disorder), (firefighters OR firemen OR fire fighters OR fire service OR firefighting), (military OR veterans OR soldiers OR armed forces), (predictors OR risk factors OR causes OR predisposition OR determinants OR cause), and (prevalence OR incidence OR epidemiology OR frequency OR occurrence OR statistics). The characteristics and results reported in each included article are described and the information is summarized in detail in the PRISMA flow diagram (Figure 1).

## 4. Results

### Characteristics of Studies

The search strategy identified a total of 1799 studies from the electronic databases searched using Covidence software (Covidence.org: Melbourne, VIC, Australia). The Covidence software automatically screened and removed 163 studies as duplicates. The remaining items (1636) were screened against the eligibility criteria set by the authors based on the title and abstract only, yielding 66 remaining records for full-text screening. Thirty-four studies were excluded in the full-text screening phase, leaving a final pool of 32 studies that were eligible for inclusion in this scoping review.

**Figure 2:**
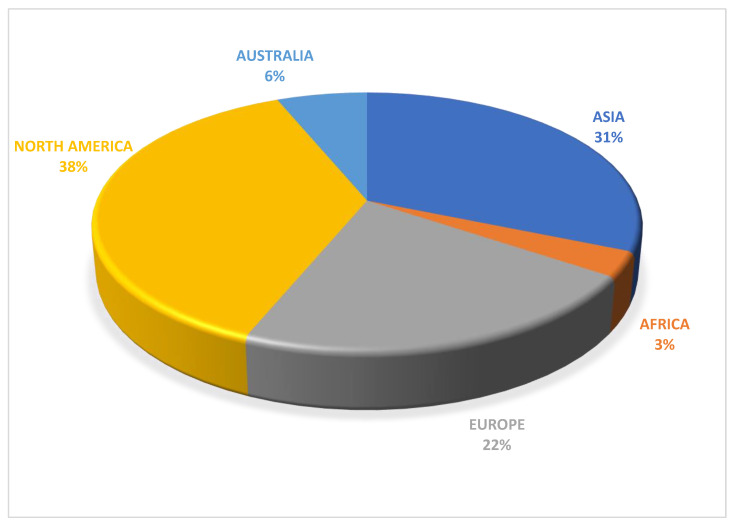
Summary of continents selected for the review.

The thirty-two studies included a total of 306,173 subjects. This scoping review included studies from 2005 to 2021. The majority of studies (75%) were published in the last ten years (2011 to 2021), 25% were from 2005 to 2010, and seven studies were from 2005–2009. Most of the studies were conducted in North America (38%), Asia (31%), and Europe (22%), while African and Australian studies represented 3% and 6%, respectively (Figure 2). In total, 50% of the studies examined firefighters, while the other 50% examined military personnel.

The samples of these studies mainly consisted of firefighters and military veterans and soldiers. The sample sizes ranged from *n* = 43 [33] to *n* = 31,534 [34]. The PTSD Checklist, Civilian Version (PCL) [35] is a 17-item self-report measure of PTSD symptoms. The items assess the individual’s experience of PTSD symptoms over the past month. The PCL has two versions: the PCL-M (reexperiencing symptoms explicitly written for military) and the PCL-C (reexperiencing symptoms written generically to apply to any traumatic event) [35]. The PCL-M version that measures PTSD symptom severity comprises 17 self-report items rated from 1 to 5 points on a Likert-type scale [36]. A cut off starting from 50 or higher and the presence of the symptoms suggest a moderate or high level per the DSM-IV algorithm for diagnosing PTSD, which is commonly used to screen for PTSD [37].

Other studies have used the Impact of Event Scale-Revised (IES-R) Korea version, comprising 22 items in Korean for self-reported PTSD symptoms experienced in the past seven days related to a specific stressor. The symptoms include intrusion, numbing, avoidance, and hyperarousal. Total scores range from 0 to 88, with PTSD positivity being indicated by a score of 18 or higher [38,39]. Similarly, other authors adopted the German Version of the Post-Traumatic Symptom Scale, with values above 12.5 being associated with a suspected diagnosis of PTSD [40]. The Japanese version of the IES-R comprised 22 items, with scores of 25 or more indicating PTSD [41]. One of the articles used in this review assessed PTSD with the SRIP, a Dutch-validated and reliable self-administered questionnaire [42]. This questionnaire consists of 22 questions, with higher score indicating more PTSD symptoms (range 22–88) [42].

PTSD in DSM-V is categorized under Trauma- and Stressor-Related Disorders, a group focused on behavioral symptoms, including four distinct diagnostic clusters instead of three; “re-experiencing, avoidance, negative cognitions and mood, and arousal” [43]. On the other hand, the DSM-IV criteria classify PTSD as an anxiety disorder. DSM-5 requires one re-experiencing, one avoidance, two cognition- and mood-related, and two arousal- and reactivity-related symptoms out of 20 qualifying symptoms. Moreover, researchers adopting either DSM-IV or DSM-V need to consider symptoms lasting at least one month and impaired functioning. To use ICD-10 criteria, individuals must exhibit one re-experiencing symptom, one avoidance symptom, and one feeling of continued threat symptom out of 17 qualifying symptoms [43]. The majority of the studies used in this scoping review adopted a pre-existing PTSD checklist either based on DSM IV, DSM V, or ICD 10/11. Table 1 and Table 2 outline the factors examined by each of the 32 included studies. The prevalence rates of PTSD among firefighters ranged from 1.9% [44] to 57% [45], while for military personnel the rates ranged from 3.72% [46] to 37.8% [36]. The study instruments used to measure the level of PTSD and its predictors also varied. Relevant and detailed methodological information was extracted and summarized from the various studies and is presented in Table 1 and Table 2.

## 5. Predictors for PTSD

### 5.1. Demographics Factors

The relationship between demographics and PTSD has been examined in some studies. For example, the age at which one starts work as a firefighter, as well as the age of the military personnel at the time of deployment to war, affects the development of PTSD [21,39,50,56]. Furthermore, marital status [67] and female sex [40] among these cohorts have been found to influence the occurrence of PTSD. Some studies showed that educational level influences PTSD, while other studies found conflicting results, for example for military personnel deployed to Iraq with higher academic qualifications [56] and for firefighters whose majors in education were information communication and equipment safety [66]. On the contrary, Meyer et al. (2012) explained that lower educational status might expose firefighters to an increased risk of developing PTSD [64].

### 5.2. Organization Factors

The hierarchy of rank in service is associated with PTSD, with soldiers and sergeants demonstrating higher levels of PTSD than officers [44]. The duration of service was also associated with PTSD; the longer one stays in service, the more exposed one is to experiencing PTSD [39,40,47,63]. Some studies examined organizational or job stress as a contributor to experiencing PTSD in both firefighters and military personnel [61,62]. Higher occupational stress [64] and burnout [60] were significant factors related to work-related PTSD. Exposure to traumatic events at work or due to one’s job description [21,50,51,52,63] and the exposure of military personnel deployed to other countries during combat [34,36,46,47,48,49,53,58] were the most significant predictors in these studies. Moreover, Soravia et al. (2021) suggested that even previously experienced work-unrelated trauma can predispose firefighters to PTSD [40]. Another study showed an association between injuries received prior before being employed in the profession [54,57] and PTSD.

### 5.3. Comorbidity

The development of PTSD cannot be mentioned without psychological disorders being mentioned, since these play a vital role in the development of PTSD [5]. Some studies revealed that pre-existing mental health conditions such as anxiety disorder [45] and depression [26,62], among other mental health comorbidities, can predict PTSD among firefighters [21,44,67] and military personnel [54,57]. However, other studies have suggested that physical illnesses also contribute to PTSD among these groups [47,67]. Poor health function [36] and concurrent conditions and behaviors, such as respiratory symptoms, exercise, and alcohol use, also play important roles in contributing to PTSD symptoms [65]. Specifically, pre-deployment nightmares among military personnel are associated with an increased risk of developing PTSD [42].

### 5.4. Social Support

Social support is a significant predictor of the development of PTSD [5]. Psychosocial stressors in the life of military personnel and firefighters have been shown to increase the risk for incurring PTSD. Tracie et al. (2013) explains that life and family stress during the deployment of military personnel [48,53] and lower social support [44,53,64] are triggers for PTSD, while post-deployment social support is a protective factor [48]. Iversen et al. (2008) found low morale and non-receipt of a homecoming brief (psychoeducation) to be triggers for PTSD [46]. Passive coping [45] and dysfunctional cognitive coping strategies [40,61] are associated with PTSD.

### 5.5. Personality

Individual personality characteristics may predict PTSD. Chung et al. (2015) showed a significant association between masculinity–femininity personality and social introversion with PTSD among firefighters [39]. Heinrichs et al. (2005) suggested that the combination of a pre-existing condition, increased hostility, and low levels of self-efficacy are strong predictors of the development of PTSD symptoms in the high-risk population of firefighters [33].

## 6. Discussion

Firefighters and military personnel are considered to work in hazardous and stressful occupations. They may be exposed to both direct and indirect stressors, such as risking one’s own life when entering a burning building, combat and wars, and witnessing the suffering of others. PTSD prevalence rates reaching 57% for firefighters and 37.8% for military personnel have been described in these groups [36,46].

The studies included in this review were acceptable in quality. The studies revealed that the varying factor increases the risk of developing PTSD among firefighters and military personnel. Based on this review, we identified that traumatic events, occupational factors, social support, physical and psychological factors, and individual traits were the main predictors of PTSD.

To our knowledge, this is the first scoping review study focusing on predictors of PTSD among firefighters and military personnel and estimating the PTSD prevalence in these populations. When the nature and intensity of duty-related exposures are considered, among other factors, the risk of developing PTSD is apparent. These studies found that prevalence rates of PTSD in this cohort exceeded those of the general population [68]. This suggests a need for targeted efforts to mitigate the risk of developing PTSD amongst firefighter and military personnel, either by addressing individual characteristics that predispose individuals to the risk, screening those going into these professions for vulnerability factors, or reducing the impacts of trauma exposure on the psyche.

## 7. Prevalence of PTSD among Military Personnel and Firefighters

Prevalence can be described as the proportion of individuals in a society with a particular disorder at a specific time. Prevalence estimates are governed by various factors of the disease, the duration of the condition, demographic characteristics, and others [23].

Prevalence is dynamic and may vary with population, place, and time [23]. Various studies have attempted to estimate the prevalence rates of PTSD among firefighters and military personnel. Prevalence estimates for PTSD rates among military personnel ranged from 2% to 17% [13]. A study among professional firefighters in Germany approximated the PTSD rate at 18.2% [69]. On the contrary, our present scoping review found high figures for both military personnel and firefighters.

Previous studies conducted after the conclusion of hostilities and conflicts in Iraq and Afghanistan have broadened the understanding of PTSD among military personnel after deployment [70]. Richardson et al. (2010) estimated the point prevalence during this time as ranging from 4% to 17% [13], while the US military recorded the highest prevalent rate during the study [13,36]. Jakupcak et al. (2008) found the prevalence rate of PTSD for US veterans deployed to Iraq or Afghanistan after a retrospective study to be 37.8%. In reverse, a rate of 3.72% was recorded in a cross-sectional study of 4762 UK military personnel deployed to Iraq [36].

Similarly, firefighters’ prevalence rates for PTSD symptoms also vary from 6.5% to 37% [5,6,45,71]. The sample size used to study the firefighters may influence the prevalence rate [3,5], along with the research participants, whether other cohorts were investigated in addition to a different cohort [45], and the scales adopted in measuring PTSD [72]. Some scales may depict incomplete measures of PTSD symptoms compared to the DSM [73].

Finally, the exact prevalence estimates of PTSD in military and firefighter populations are not known. Therefore, it is not necessary to bother with the precise prevalence rates [70] for these populations. The big challenge is ensuring that PTSD is acknowledged swiftly and that reliable pathways to evidence-based care are available [70].

## 8. Predictors of PTSD among Military Personnel and Firefighters

### 8.1. Demographic Factors

Sociodemographic variables may contribute to PTSD among firefighters and military personnel. Previous studies have distinguished certain demographic predictors that increase the occurrence of PTSD, viz. gender, age, educational status, marital status, and socioeconomic status [21,50,74]. Specifically, the impacts of younger age during traumatic events and exposure to PTSD are difficult to predict. Moreover, there are conflicting effects on the generation and processes of traumatic stress reactions [75,76].

Our review of three studies examined age as a risk factor for PTSD in both military personnel and firefighters [21,39,50]. A previous study suggested that having a younger age at the time of trauma is primarily unrelated to PTSD [77]. Forbes et al. (2016) also suggested that age at the time of deployment of the military personnel was related to experiencing PTSD [50]. The same research group revealed that military personnel who were older at deployment are more likely to have current PTSD [50]. On the contrary, other studies have shown that younger personnel were about four times more likely to experience PTSD than the older age group, with a steadily decreasing age effect [78]. Firefighters advanced in age may have been more exposed to traumatic events than younger ones [39].

Over the past 20 years, the number of males serving in the military and the fire service has outnumbered the number of females [79,80]. In 2018, females made up about 8% of the fire service [79]. The number of females has increased over twenty years now, and this number is estimated to increase further [77]. Consequently, this projected increase in the female gender puts females at a high risk of experiencing mental illnesses, including PTSD [77,80]. A cross-sectional study in Switzerland suggested that female firefighters are at risk of PTSD [40], while additional factors may contribute. However, a concrete reason may be that women in the military are inadequately prepared [81]. On the other hand, female fighters experience physical, emotional, and occupational stressors [82] with higher rates of depression, which predisposes them to PTSD. Surprisingly, only one article [40] that met the criteria for inclusion in this scoping review examined relations between female gender and PTSD. Despite the studies included, conclusions cannot be made on gender and PTSD among firefighters and military personnel. A meta-analysis involving 32 articles revealed that military personnel with higher education levels might adopt better coping strategies than those with lower educational levels [77]. Similarly, Meyer et al. (2012) suggested that firefighters with lower educational levels risk PTSD [64]. Notwithstanding, these findings differed from Rona et al. (2012), who found that the higher educational level of the military personnel, the more they are exposed to PTSD [56].

In other studies, demographic characteristics such as employment status have been shown to affect PTSD. Regarding the lifetime prevalence of PTSD, being employed has been shown to reduce the occurrence of PTSD by half compared to the unemployed [78]. Likewise, unemployed individuals after military training are more likely to exhibit PTSD symptoms [77]. Individuals with single marital status are at risk of PTSD. One article showed that being single as a firefighter put individuals at risk of PTSD compared to being married, since they may have reduced emotional and social support [45].

### 8.2. Job Factors

Job and organizational stress [36,47,62], burnout [60], length of service [39,40], rank in service [44], and traumatic events [48,49,50,61] were found to be associated with increased risk of PTSD among military personnel and firefighters. Within organizations, it is said that non-experts are at risk of PTSD symptoms [83]. Soravia et al. (2020) studied first responders and the risk of developing PTSD, with the findings showing that length of service is related to PTSD symptoms [40]. Higher job rank levels (soldiers and sergeants) among firefighters showed a significant association with PTSD [44]. An explanation may be that as one advances in their career, more experience is acquired, and more traumatic events are encountered, exposing one to PTSD.

Exposure to traumatic events is the most prevalent factor for developing PTSD among military personnel and firefighters. Deployment to war and combat zones makes military personnel vulnerable compared to those not deployed to war zones [34]. Another study showed that being deployed to a ‘forward’ area during combat makes the individual more at risk compared to colleagues not at the front [46]. Furthermore, combat roles during deployment to Iraq or Afghanistan were associated with significantly acute mental health outcomes [58]. A cross-sectional study among various rescue teams with a cohort of 239 firefighters showed that work-related trauma was a predictor of PTSD [40]. Military personnel discharge weapons or witness injury and death during deployment [77]. These memories of trauma and intense fear increase their risk of PTSD [49]. Stevelink et al. (2018) studied 10,272 military personnel deployed to Iraq or Afghanistan and concluded that military personnel in combat roles developed worse mental health outcomes [58]. Among firefighters who experience elevated levels of job-related trauma, there are significantly higher risks of PTSD and chronic PTSD [63], while non-work-related trauma significantly predicted only chronic PTSD but not PTSD [63]. The category and intensity of the traumatic events may likely be the risk factors correlated with the differences in PTSD in firefighting [21]. These findings are consistent with Trickey et al. (2013), suggesting that the intensity of the trauma is associated with the likelihood of PTSD [84]. Despite the complexity of the traumatic exposure, estimating the conceptual factors is tricky [84]. Studies among firefighters suggest that higher organizational stress increases PTSD [39,62,64]. Nonetheless, occupational stress is not only associated with PTSD, but also with depression and alcohol abuse [85]. Continuous exposure to traumatic events has been shown to negatively affect one’s psychological health, with increased flashbacks and irritability [86]. In our study, few articles supported perceived stress and general stress as predictors of PTSD among firefighters and military personnel [26,44]. Burnout has been recorded as a factor for PTSD. In a study in South Korea, burnout was shown to correlate with PTSD among firefighters [60]. Another study categorically showed that the emotional exhaustion of burnout is directly linked to PTSD [72], while countermeasures were proven as protective factors for PTSD [72]. On the flip side, a greater number of years in service is linked to burnout and the risk of PTSD [85].

### 8.3. Social Support

Social support is defined as information an individual receives in the form of believing they are loved and cared, for while feeling esteemed and affiliated with a network of social and mutual obligations [5]. Tracie et al. (2013) explained that general life stress and family stress during deployment of military personnel increase the likelihood of PTSD [48]. Firefighters and military personnel are exposed to a high degree of stress at work. Therefore, they require support from their family and friends to reduce the impact of this stress on them. According to a study by Rona et al. (2012), social support is a significant predictor for PTSD, as it plays a mediating role between traumatic events and other mental health conditions [56]. Furthermore, among the military personnel, low morale and poor social support within the unit and non-receipt of a homecoming brief, i.e., psychoeducation [46], augment the development of PTSD. Another study supports these findings. The literature shows that UK military personnel deployed to Iraq may have PTSD due to feeling unsupported on return from deployment [56,64]. This supports previous findings indicating that perceived social support is likewise a predictor of PTSD symptoms among professional firefighters and military personnel exposed to various traumatic events [36,41,43,49]. The findings from previous studies suggest that good social support acts as a buffer against the adverse outcome of traumatic stressors among PSPs [17]. Overall, high social support will positively affect self-reliance and self-security and serve as a protective factor [77].

### 8.4. Psychological and Physical Disorders

Few studies have ascertained the relationship between psychological and physical illness as a risk factor for PTSD among firefighters and military personnel, in which traumatic exposure takes the lead. However, it is difficult to ascertain the effects of mental and physical health on the occurrence of PTSD. Notwithstanding, some studies have revealed that some psychological and physical conditions predispose military personnel or firefighters to PTSD [36,45,47,57,67], such as depression and anxiety. Likewise, a longitudinal study among rescue personnel showed that workers exposed to traumatic events had significantly higher depression levels than less-exposed workers [87]. Nightmares before deployment have also been associated with an increased risk of PTSD among military personnel [42]. A study showed that anxiety and depression comorbidities are usually noticed among firefighters and military personnel diagnosed with PTSD who had concurrent psychological conditions [88,89]. These findings show PTSD as consistently coexisting with other mental health conditions [90]. Wang et al. (2011) examined 1056 Chinese military personnel who assisted in an earthquake rescue operation and concluded that personnel who have not received psychological counseling and who habitually abuse alcohol are at risk of PTSD [52]. Additionally, other studies have shown that having a diagnosis from a mental health professional [26], previous history of psychological treatment, and non-receipt of any psychological counseling [52] increases the risk of developing PTSD. Soo et al. (2013) linked those concurrent conditions and behaviors, such as respiratory symptoms, exercise, and alcohol use, to PTSD symptoms [65].

### 8.5. Injuries

Few studies have illustrated a relationship between PTSD and physical injury. Ozer et al. (2003) revealed that psychological processes during the traumatic event are the most significant predictors of PTSD [91]. A study examining injury and PTSD among US military personnel showed that injuries sustained during battel were actively associated with PTSD compared to non-battle injuries [57]. Sandweiss et al. (2011) added that irrespective of the severity of injury sustained, self-reported preinjury was significantly associated with PTSD [54]. Occupational injuries result in physical disabilities and psychological disturbance, worrying injury memories, impairment of contextual memory, emotional disorders, and PTSD [92]. A study to determine the effects of occupational trauma on individuals’ psychological ability revealed that among those who had an injury at work, 12% had PTSD. In comparison, 11% had subsyndromal PTSD after six months of follow-up assessment [93].

### 8.6. Other Factors

The coping strategy has been identified as another important risk factor for developing PTSD among firefighters and military personnel. A longitudinal study among firefighters provided in-depth knowledge of coping behaviors to aid function after traumatic events [21]. Different coping styles are associated with PTSD. For example, females who are very sensitive to threats are less likely to use effective coping strategies [77]. Passive coping strategies [45], problem- and emotion-focused coping, and mixed focused coping [94] were found to relate to experiencing PTSD among military personnel and firefighters. Sattler et al. (2014) demonstrated that disengagement coping [95] and self-blame coping were associated with PTSD [64]. Some personality traits of individuals who experience repeated traumatic exposure may predict PTSD [39]. Chung et al. (2015) found that masculinity–femininity and social introversion traits expose one to PTSD [39]. Another study examined certain personality traits, which revealed that neuroticism was linked to PTSD [96]. A neurotic individual is sensitive to the stressor, and their responses are fast and intense, yet slow to return to a normal state [96].

## 9. Trajectory of PTSD among Military Personnel and Firefighters

The trajectories for PTSD symptoms among people exposed to traumatic events are complex [97]. PTSD is categorized in the DSM and ICD as an anxiety disorder and one of neurotic, stress-related, and somatoform disorders [70]. The first criterion to be met in both systems is that the individual experiences a severe stressor, primarily a severe threat to life or physical integrity, i.e., the individual experiences or witnesses a traumatic event, such as combat, a natural disaster, fires, or a violent personal assault.

Furthermore, both the DSM and ICD require evidence of experiencing the trauma in the form of intrusive thoughts, nightmares, or dissociative flashbacks, resulting in severe stress via these reminders. More often than not, these are usually symptoms of traumatic stress and a focus of interest [70]. The re-experiencing symptoms may be the distinctive trademark of traumatic stress, manifesting as either poor prognosis, chronic PTSD, or disabling PTSD [98]. Finally, PTSD diagnosis requires a cluster of intrusion, avoidance, and arousal symptoms. Significantly, this condition in the past and present members of the military and firefighters is linked to infirmity, poor quality of life, and increased physical health problems [99,100]. Studies have shown that about 80% of individuals with PTSD meet the criteria for other mental health conditions such as substance use disorder or anxiety [101,102]. Paradoxically, these comorbidities may just be a result of inadequate symptom specification at diagnosis [103]. A study by Kessler et al. (1995) revealed that about one-third of individuals who experience an episode of PTSD never recover, even after many years [102]. Regarding the course, PTSD in military personnel and firefighters, both in active service and as veterans, becomes chronic to the condition [104]. For example, a prospective longitudinal study of 214 veterans from 1982 in Lebanon showed that the rate of PTSD was reduced three years after war but then increased 17 years after war [104]. The delayed onset of PTSD is one debatable trajectory of the condition. Usually, PTSD occurs at any age, beginning after the first year of life, with symptoms initiating within the first three months after exposure to the trauma [9]. Delayed PTSD is when the disorder surfaces at least six months after the traumatic exposure [9,70]. Andrews et al. (2007) showed that the delayed onset of PTSD is seldom seen in civilian populations but may be more common in veterans [105]. Consequently, a longitudinal study among firefighters revealed that delayed onset during follow-up was linked to functional impairment [106].

## 10. Implications for Public Policy and Practice and Future Directions

This study reveals that the prevalence rates of PTSD among military personnel and firefighters are high compared to reported rates in the general population. Early interventions are vital in preventing PTSD among firefighters and military personnel. This study also highlights vital predictors of PTSD among military personnel and firefighters. PSPs require adaptation skills and trauma management at work to reduce the occurrence of PTSD and increase productivity. Health policy directors and managers need to integrate interventions that mitigate vulnerability factors in PSP and increase their resilience as a way of preventing PTSD in members of the military and firefighters. Leaders in these cohort and the community can also provide continuous support to reduce stress and PTSD [77]. Additionally, psychological interventions have been proven to reduce PTSD. While interpersonal psychotherapy and cognitive–behavioral therapy can be effective, long waiting times are expected [107]. Despite the increase in PTSD among military personnel and firefighters, most individuals do not seek help from health professionals nor report symptoms [68]. The percentage of PSPs receiving treatment for mental health illness is estimated to be significantly lower due to stigma associated with mental illness and privacy loss [68]. Notwithstanding, internet-delivered cognitive behavioral therapy may be recommended [108], and supportive text messages programs such as “Text4PTSI” and “Text4Wellbeing” can reduce mental health problems, with the former specifically reducing PTSD among first responders [109]. Various randomized controlled clinical trials have proven that supportive messaging mitigates depression, anxiety, and stress, and may mitigate PTSD [110,111,112,113,114].

Governments and policymakers need to make a conscious effort to improve mental health services among PSPs to improve their quality of life. These findings will help health professionals understand the various predictors and provide the best treatment regimen for individuals with comorbid physical and mental illnesses to reduce the risk of PTSD. Future research should focus on interventions aimed at preventing and reducing PTSD among military personnel and firefighters. Further research is also needed to examine how identified predictors interact and provide strategies to protect these cohorts and reduce the prevalence of PTSD.

## 11. Limitations

The authors of this scoping review acknowledge several limitations. Firstly, in this scoping review, we only searched English language databases. Much effort was made to identify all relevant studies for this review considering our eligibility criteria. However, we may have left out some relevant studies, especially those published in other languages. In addition, the various studies included in this review used different screening measures and international diagnostic classifications for the determination of PTSD, which could potentially lead to variations in prevalence estimates. Finally, there was no assessment of risk of bias for the included studies, which is a limitation. Notwithstanding the limitations of the study, this scoping review provides an insightful overview of the prevalence and predictors of PTSD among military personnel and firefighters.

## 12. Conclusions

This review has identified a wide range of risk factors associated with PTSD among firefighters and military personnel. As clearly illustrated, exposure to traumatic events is just one of several possible predictors. Job stress, physical and psychological comorbidities, demographics characteristics, personality traits, and social support systems similarly predict PTSD among military personnel and firefighters.

This scoping review adds to the literature implicating multiple factors in predicting PTSD among military personnel and firefighters. The present study highlights an overall prevalence of PTSD. Social interventions and effective support from the family and friends of these PSPs are needed to reduce psychological stress. Governments and policymakers should be mindful of these factors and should try to improve the mental health of these PSPs. Proactively identifying military personnel and firefighters with risk factors for developing PTSD and offering then support and treatment can reduce future psychopathology and minimize the risk of PTSD. E-mental health services such as daily supportive text messaging can be incorporated to reduce the risk of PTSD [111,112,113,114,115,116,117,118,119,120,121].

## Figures and Tables

**Table 1:** Summary of studies with prevalence and predictors of PTSD among Military Personnel.

Author and Year	Country	Study Design	Size	Occupation	Prevalence of PTSD and Measure	Predictors
Grieger T.A. et al. (2006) [47]	US	Cross-sectionaland longitudinal	243	Soldiers	28.4% (1, 4, 7 months) PTSD Checklist, Civilian Version	Physical problem Severity Combat exposure Deployment length
Jakupcak M. et al. (2008) [36]	US	Retrospective	108	Veterans deployed to Iraq/Afghanistan	37.8% PTSD Checklist, Military Version	Poor health function Combat and chemical exposure
Tracie S.M. et al. (2013) [48]	US	Cohort	238	National Guardsoldiers	12.60% DSM-IV-TR	Combat exposure Life and family stress during deployment Post-deployment social support
Connell M.A. et al. (2013) [49]	South Africa	A cross-sectional and descriptive study	1527	Veterans	33% Impact of Event Scale—Revised	exposed to combat during the border war
Forbes D. et al. (2016) [50]	Australia	Retrospective study	1025	Veteran peacekeepers deployed on one or more of seven UN-sanctioned interventions between 1989 and early 2002.	16.8% World Mental Health Survey Initiative version of the World Health Organization’s Composite International Diagnostic Interview, Version 3.0 (CIDI)	Age at the time of deployment was Traumatic life events exposure In terms of overall life experiences, the most common PTEs were Transport accidents physical assaults and the sudden unexpected death of someone close
Zamorski M. et al. (2016) [51]	Canada	Longitudinal	2002 and 2013 (n = 5155 and 6996, respectively)	Armed forces	DSM-IV 5.3%	Traumatic exposure
Wang H. et al. (2011) [52]	China	Retrospective	1056	Military	6.53% Davidson Trauma ScaleEarthquake Experiences Scale	Traumatic exposure/earthquake experiencenot having received psychological counseling regular drinking
Liu B. et al. (2016) [53]	China	Cohort study	303	Veterans	29.0% The Post-Traumatic Stress Checklist—Civilian Version	Combat exposure Social support and family-disclosure norms
Sandweiss D.A. et al. (2011) [54]	USA	Cohort	22,630	Military personnel deployed to Iraq and Afghanistan	8.10% DSM-IV	Self-reported preinjury Psychiatric status
Hu Y. et al. (2020) [55]	USA	Cross-sectional	1042	Veteran	7.1% PTSD Checklist	Current Depression Current insomnia Concussion Low social support
Iversen A.C. et al. (2008) [46]	UK	Cross-sectional	4762	Military personnel deployed to Iraq	3.72% PTSD Checklist	Deployed to a ‘forward’ area Low morale and poor social support within the unit and non-receipt of a home-coming brief (psycho-education)
Macera C.A. et al. (2014) [34]	USA	Cohort	31,534	Military personnel deployed to Afghanistan or Iraq	5.38% DSM-IV	Combat exposure or other deployment-related characteristics
Rona R.J. et al. (2012) [56]	UK	Longitudinal Cohort study	6292	Military personnel Iraq	3.90% PTSD Checklist, Civilian Version (PCL-C)	Higher educational qualification Feeling unsupported on return from deployment Deployed not with parent unit Multiple physical symptoms Perception of poor or fair health Older age and Perception of risk to self
Macgregor A.J. et al. (2013) [57]	USA	Cohort	1777	Military personnel	25.15% ICD-9	Previous mental health diagnosis within 1 year of deployment Previous Battle injury
Van Liempt S. et al. (2013) [42]	Dutch	Prospective longitudinal cohort	453	Military personnel deployed to Afghanistan	6.6% Self-Rating Inventory for PTSD (SRIP)	Existence of pre-deployment nightmares is associated with an increased risk for the development of PTSD
Stevelink S.M.A. et al. (2018) [58]	UK	Cohort study	10,272	Military personnel	6.2% PTSD Checklist (PCL-C)	Deployment to Iraq or Afghanistan and a combat role during deployment were associated with significantly worse mental health outcomes and alcohol misuse in ex-regular personnel but not in currently serving regular personnel.

PTDS—Post Traumatic Stress Disorder, DSM—Diagnostic and Statistical Manual of Mental Disorders, ICD—International Classification of Diseases.

**Table 2:** Summary of studies with prevalence and predictors of PTSD among Firefighters.

Author and Year	Country/Religion	Study Design	Size	Occupation	Prevalence of PTSD and Measure	Predictors
Del Ben K.S. et al. (2006) [21]	US	Cohort	131	Firefighters	8% PTSD ChecklistImpact of Event Scale	Previous psychological treatment Age at which one started work Miscellaneous Calls Response of horror following the firefighter’s Single Worst Event
Na K.S. et al. (2017) [59]	Korea	Cross-sectional	507	Firefighter	27.4% Impact Event Scale-Revised Korean Version	Age Duration of service Marriage Depression
Alghamdi M. et al. (2016) [45]	Saudi Arabia	Randomized, controlled	200	Firefighters	57% Screen for Post-traumatic Stress Symptoms (SPTSS)DMS-IV	Anxiety Depression Passive coping strategies
Heinrichs M. et al. (2005) [33]	Germany	Prospective Follow-Up Study	43	Male firefighters	At 24-month follow-up, 16.3% met the criteria for PTSD and 18.6% subsyndromal PTSD according to the PTSD Symptom Scale	Preexisting high levels of hostility Low levels of self-efficacy Personality traits
Jo I. et al. (2018) [60]	South Korea	Retrospective	109	Firefighters	Full PTSD criteria was 2.7% and partial PTSD was 2.7%, Thus, 5.4% of the participants were in high risk of PTSD. Impact of Event Scale-Revised-Korean version	Burnout
Armstrong D. et al. (2014) [61]	Australia	Cross-Sectional Study	218	Firefighters	23% Impact of Events Scale-Revised (IES-R)	Organizational stress Traumatic events Job stress Cognitive coping
Saijo Y. et al. (2012) [62]	Japan	Cross-sectional	1621	Firefighters	9.7% Japanese version of the Impact of Events Scale-Revised (IES-R)	Depression Job stress Social support
Langtry J. et al. (2021) [63]	UKIreland	Cross-sectional	1300	Firefighters	Complex PTSD criteria were met by 18.23% and PTSD criteria were met by 5.62% of the sample International Trauma Questionnaire ICD-11 criteria	Experiencing higher levels of service-related trauma significantly increased the risk for both PTSD and CPTSD, and non-work-related trauma uniquely predicted CPTSD but not PTSD.
Chung I. S. et al. (2015) [39]	Korea	Cross-sectional	185 male firefighters	Firefighters	35.1% Impact Event Scale-Revised Korean Version (IESR-K)	Job duration Age Masculinity-femininity (Personality) Social introversion (Personality) Job stress
Meyer E.C. et al. (2012) [64]	USA	Cross-sectional	142	Firefighters	6.4% The PTSD Checklist-Civilian	Lower education Low Social Support Higher Occupational Stress
Noor N. et al. (2019) [26]	USA	Retrospective	75 female and 2564 male	Firefighters	Twenty per cent of the women and 12% of men reported relatively high levels (≥39) of PTSD symptoms. The PTSD Checklist—Civilian Version	Depression Having seen mental health professional General stress
Shi J. et al. (2021) [44]	China	Cross-sectional	261	Firefighters	1.9% PTSD Checklist for DSM-5	Perceive stress Social support Rank (Soldiers and sergeants)
Soravia L.M. et al. (2020) [40]	Switzerland	Cross-sectional	239	Firefighters	8% German Version of the Post Traumatic Symptom Scale (PTSS-10)	Female sex Previously experienced work-unrelated trauma Work-related trauma Years on the job Dysfunctional coping strategies Problem-focused coping strategies and self-efficacy
Soo J. et al. (2011) [65]	USA	Longitudinal	11,006	Exposed World Trade Center—Firefighters	7.4% PTSD Checklist	Concurrent conditions and behaviors, such as Respiratory symptoms, exercise, and alcohol use also play important roles in contributing to PTSD symptoms.
Sun X. et al. (2020) [66]	China	Cross-sectional	409	Firefighters	4.89% PTSD checklist for DSM-5	Firefighters whose majors were InformationCommunication and Equipment Safety reported higher levels of depressionand PTSD
Chen Y.S. et al. (2007) [67]	Taiwan	Two-stage survey	410	Firefighters	10.5% PTSD Checklist	Mental status Psychosocial stressors, or perceived physical condition

PTDS—Post Traumatic Stress Disorder, DSM—Diagnostic and Statistical Manual of Mental Disorders, ICD—International Classification of Diseases.

## Data Availability

Not applicable.
